# Children and Adolescents’ Susceptibility to Domoic Acid in Southern China: Preliminary Evidence Revealing Baseline Exposure Profiles and Multidimensional Influencing Factors

**DOI:** 10.3390/toxics13080628

**Published:** 2025-07-26

**Authors:** Yuxin Lin, Tingze Long, Siyi Zou, Rui Hua, Meixia Ye, Shengtao Ma, Bo Peng

**Affiliations:** 1 Department of Pharmacy, College of Pharmacy, Jinan University, Guangzhou 511443, China; lyxinjnu@stu2022.jnu.edu.cn; 2 School of Pharmacy, Guangdong Pharmaceutical University, Guangzhou 510006, China; longtingze123@gmail.com; 3 Reproductive Medicine Center, Department of Obstetrics and Gynecology, Nanfang Hospital, Southern Medical University, Guangzhou 510515, China; zsy150070077@smu.edu.cn (S.Z.); ruih218@163.com (R.H.); 4 School of Public Health, Guangzhou Medical University, Guangzhou 511436, China; misha@gzhmu.edu.cn (M.Y.); mast@gzhmu.edu.cn (S.M.); 5 Key Laboratory of Philosophy and Social Science in Guangdong Province of Community of Life for Man and Nature, College of Environment and Climate, Jinan University, Guangzhou 511443, China

**Keywords:** domoic acid, biomonitoring, DNS-Cl derivatization, HPLC–MS/MS, urine samples

## Abstract

Domoic acid (DA) is a potent neurotoxin that poses public health concerns, especially for children and adolescents during critical neurodevelopmental periods. In the present study, urinary DA concentrations in 216 children and adolescents at the age of 6 to 18 in southern China were determined using a novel dansyl-chloride (DNS-Cl) derivatization high performance liquid chromatography–tandem mass spectrometry (HPLC–MS/MS) method with ultrahigh sensitivity (LOQ: 0.087 ng/mL). The median urinary DA concentration was 2.17 ng/mL (interquartile range (IQR): 0.87–4.08 ng/mL). When analyzed by age group, the medians were 1.40 ng/mL (6–9 years; IQR: 0.55–3.49 ng/mL), 2.16 ng/mL (10–13 years; IQR: 0.94–4.07 ng/mL), and 2.93 ng/mL (14–18 years; IQR: 1.06–5.06 ng/mL). Our findings revealed that urinary DA concentrations increased with age and varied significantly across different body mass index groups (*p <* 0.05), while no significant gender differences were observed. The estimated daily intake (1.73–374 ng/kg/day) remained below established safety thresholds. This study represents the first systematic biomonitoring of urinary DA exposure in children and adolescents from southern China’s coastal communities, addressing critical knowledge gaps and establishing baseline data amid rising harmful algal bloom frequency.

## 1. Introduction

Domoic acid (DA) is a naturally occurring marine neurotoxin produced primarily by certain species of diatoms, notably those in the genus *Pseudo-nitzschia*. It bioaccumulates in shellfish and finfish, entering the marine food web and posing significant risks to both marine organisms and humans [1]. Structurally similar to glutamate, DA acts as a potent agonist for α-amino-5-methyl-3-hydroxy-4-isoxazolepropionic acid (AMPA) and kainate glutamate receptors [2]. This interaction triggers excessive calcium influx, leading to excitotoxicity and neuronal damage, particularly affecting developing neurons [3].

Acute exposure to DA in humans can cause amnesic shellfish poisoning, characterized by gastrointestinal symptoms such as vomiting and diarrhea, neurological effects including confusion, memory loss, and seizures, and in severe cases, coma or death [4]. Beyond acute toxicity, chronic low-dose exposure to DA represents a growing public health concern [5], especially as climate change and water pollution increase the frequency and geographic range of harmful algal blooms that produce DA [6,7,8].

Preclinical studies have demonstrated that domoic acid exposure can induce a spectrum of neurotoxic effects, including seizures, memory impairment, neuronal loss (particularly in the hippocampus), and behavioral alterations such as tremors and social withdrawal [9]. Additionally, DA has been shown to cause oxidative stress and DNA damage in animal models, underscoring its potential for long-term neurological and cytogenetic harm [10]. Children and adolescents are particularly vulnerable due to a combination of factors: a more permeable blood–brain barrier [11], immature renal clearance leading to prolonged retention of toxins [12], and greater susceptibility to injury during the critical process of neurodevelopment [13].

In China, the widespread distribution of DA-producing algae in coastal waters presents a significant and growing hazard to marine ecosystems and public health [14]. Harmful algal blooms in Chinese waters have resulted in significant DA contamination of shellfish [15]. For instance, recent monitoring has consistently detected DA in shellfish along China’s coastline, with one comprehensive survey finding contamination in 43.9% of samples and concentrations up to 943 μg/kg (wet weight) [16]. This contamination can easily enter the human food chain due to bioaccumulation of toxins by filter-feeding organisms such as clams, oysters and mussels [4]. Humans are primarily exposed to DA through the consumption of these contaminated shellfish [17]. Although the regulatory limit for shellfish (20 mg/kg) is effective in preventing acute toxicity events, it does not circumvent the health risk issues associated with low-dose DA exposures [18,19]. Although available studies have not reported exceedances of regulatory limits in Chinese shellfish, the habitual consumption of seafood among coastal populations in China creates a risk of long-term, low-dose DA exposure, the health implications of which are poorly understood [20].

Animal studies have demonstrated that susceptibility to DA neurotoxicity varies with age and sex, with older rats showing increased sensitivity to DA neurotoxicity and males exhibiting greater susceptibility than females [21,22]. It is imperative to quantify human exposure and investigate these influencing factors in vulnerable populations.

However, current understanding of the health impacts of chronic low-dose DA exposure in humans is limited by a predominant focus on acute intoxication in regulatory frameworks, technical challenges in detecting DA in biological samples, and a lack of longitudinal biomonitoring data in affected coastal communities [23]. Urinary DA is an excellent, non-invasive biomarker of recent exposure, as the toxin is rapidly excreted unchanged via glomerular filtration [24]. The methodology using dansyl-chloride derivatization combined with liquid chromatography–tandem mass spectrometry (LC–MS/MS) gave us new ideas and also confirmed the feasibility of detecting trace levels of DA in biological matrices [25,26]. However, the method is still not able to meet our needs for the detection of DA in human urine. Establishing baseline urinary DA levels is therefore a critical first step toward identifying high-risk demographics and quantifying health effects.

To address these critical knowledge gaps, we aim to develop and validate a novel, ultra-sensitive high-performance liquid chromatography–tandem mass spectrometry (HPLC–MS/MS) method employing dansyl-chloride (DNS-Cl) derivatization for the quantification of DA in human urine. We hope to implement this methodology to conduct the first systematic biomonitoring of DA exposure in a long-term cohort of children and adolescents (ages 6–18) from coastal communities in southern China. This study is designed to establish the first regional exposure reference data while identifying key influencing factors, providing critical neurodevelopmental-stage-specific baseline data for assessing DA’s health impacts. Ultimately, it is hoped that these findings will help to establish an essential baseline for future studies and will help refine environmental health policies aimed at protecting neurodevelopment in vulnerable populations.

## 2. Materials and Methods

### 2.1. Chemicals and Reagents

Domoic acid was supplied by Alta Co., Ltd. (Tianjin, China), DNS-Cl was provided by Millipore Sigma (St. Louis, MO, USA). Artificial urine and 5-(Dimethylamino-d_6_) naphthalene-1-sulfonyl chloride (DNS-Cl-d_6_) were purchased from Phygene Biotechnology Co., Ltd. (Fuzhou, China). Borate buffer was bought from Shanghai Mclean Biotechnology Co., Ltd. (Shanghai, China). HPLC solvents such as acetonitrile, formic acid and Optima LC/MS grade water were offered by Shanghai Amper Experiment Technology Co., Ltd. (Shanghai, China).

### 2.2. Sample Collection

Urine samples were obtained from 216 children and adolescents aged 6–18 years during routine physical examinations at the outpatient department of Nanfang Hospital, Southern Medical University, in June 2024. Before participation, guardians provided informed consent after receiving a clear explanation of the study objectives. Each participant completed a questionnaire documenting demographic information (age, gender), dietary habits, and living environment details. Height and weight measurements were also recorded for all subjects. Mid-stream urine specimens were collected between 08:00 and 10:00 using pre-cleaned polypropylene containers. All samples were immediately stored frozen at −80 °C until laboratory analysis. The present study was approved by the Medical Ethics Committee of Nanfang Hospital (NFEC-2023-528).

### 2.3. Urine Sample Preparation

**Figure 1:**
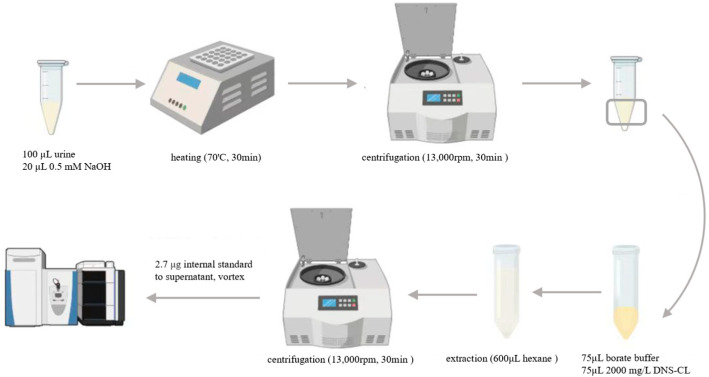
Schematic diagram of sample analysis procedure for DA.

A stock solution of 2 mg/mL DNS-Cl solution in acetonitrile was prepared and stored at 4 °C, while a 90 ng/mL DNS-d_6_-DA stock solution was freshly prepared daily. The derivatization reaction mixture was prepared by combining 30 μL of 4.5 μg/mL DA solution with 735 μL of 2 mg/mL DNS-Cl-d_6_ solution and 735 μL 500 mM borate buffer (pH = 9.5) (Figure 1).

Sample preparation began with the addition of 20 μL of 0.5 mM NaOH to 100 μL of the urine sample, followed by 5 min of vortexing. Next, the mixture was subjected to 30 min of heating at the temperature of 70 °C for precipitating proteins. The 30 min of centrifugation at 13,000 rpm was followed by the removal of the supernatant and the collection of the bottom layer for the derivatization reaction. The DA derivatization with DNS-Cl was based on a previously described method [26] with minor modifications. To the prepared bottom layer, 75 μL of 2 mg/mL DNS-Cl solution and an equal amount of 500 mM borate buffer (pH 9.5) were added and underwent 5 min of vortexing. The mixture underwent 20 min of incubation at room temperature to allow the reaction to proceed. Excess DNS-Cl was subsequently removed, which was followed by three successive extractions with 600 μL n-hexane. Finally, 30 μL of the isotope-labeled tracer was put into the remaining aqueous layer, vortexed, and analyzed directly.

### 2.4. HPLC–MS/MS Analysis

The analysis of DNS-DA and DNS-d_6_-DA was performed with an Applied Biosystems API 4000 mass spectrometer (Foster City, CA, USA) coupled with an Agilent Technologies 1260 series liquid chromatograph (Santa Clara, CA, USA). Chromatographic separation was achieved on an EC-C18 column (4.6 mm × 100 mm, 2.7 μm, Agilent, Santa Clara, CA, USA) maintained at 40 °C. The mobile phase consisted of (A) water and (B) acetonitrile containing 0.1% formic acid and 0.2% formic acid, respectively, and was delivered at 0.4 mL/min. A 30 min gradient elution was employed and the injection volume of the sample was 10 μL. The gradient program began with 5% B, rose to 99% B, maintained at 99% B and followed by column re-equilibration at 5% B for 2, 8, 12 and 8 min, respectively. Mass spectrometric detection was performed in a positive ionization mode with the following parameters: a source temperature of 500 °C, curtain gas (CUR) at 20 psi, auxiliary gases (GS1 and GS2) at 35 psi (all using nitrogen), ionization voltage (IS) of 5500 V, declustering potential (DP) of 50 V, and entrance potential (EP) of 10 V. The quantification of DNS-DA and DNS-d_6_-DA was performed using the transitions *m*/*z* 545 > 170 and 551 > 505, respectively, while qualification was achieved using the transitions *m*/*z* 545 > 453 and 551 > 177, respectively.

### 2.5. Method Validation

To prepare the calibration curve, DNS-DA standard solutions were added to deionized water to achieve ultimate concentrations that ranged from 0.1–5 ng/mL (0.1, 0.2, 0.5, 1, 2 and 5 ng/mL). The previously described method was employed to analyze these samples and assess whether the calibration curve was linear. The limit of detection (LOD) and quantification (LOQ) were obtained on the basis of signal-to-noise ratios (S/Ns) of 3 and 10, respectively. Standard DA solution was spiked into artificial urine and deionized water to assess method recovery (*n* = 3). These samples were prepared and parsed under the established HPLC–MS/MS circumstances. Recovery was calculated as the ratio of measured concentrations to spiked concentrations.

Matrix effects (ME) were assessed by comparing the response ratios of DNS-DA to DNS-d_6_-DA in spiked deionized water and pre-treated artificial urine (*n* = 3). Samples were prepared and analyzed according to the aforementioned procedure. ME was quantified by calculating the ratio of analyte-to-tracer response in the blank matrix divided by the same ratio in pure solvent.

### 2.6. Exposure Risk Assessment

The estimated daily intake (*EDI*, ng/kg/day) of DA was calculated from urinary concentrations using the following equation:

(1) $$EDI=\frac{C_{m}\times V_{urine}}{f\times bw}$$

where *C_m_* represents the unadjusted concentration of DA in urine (ng/mL). *V_urine_* is the daily urinary excretion volume (L/d), *f* denotes the proportion of DA excreted in urine relative to the total exposure dose, and *bw* is the child’s body weight (kg). Age-specific mean values for *V_urine_* were obtained from published literature for each child’s age group [27]. Rat studies have demonstrated nearly 100% urinary excretion of DA within 24 h following oral exposure [5,28]. In the absence of human experimental data, an *f* value of 100% is adopted for this study.

### 2.7. Statistical Analysis

Statistical Package for the Social Sciences (SPSS) Statistics (Version 13.0, Chicago, IL, USA) was applied to conduct statistical analysis. Samples with concentrations less than the LOD of the instrument were given a value of LOD/2. The Shapiro–Wilk test was utilized for assessing whether the urinary concentration data for each target analyte were normal. Due to the deviation of all the data from normal distribution, non-parametric tests were used: Kruskal–Wallis H and Mann–Whitney U tests were utilized for comparing the differences in individual DA concentrations across demographic variables, including age, gender and body mass index (BMI). The relationships of individual DA concentrations with demographic variables were examined by conducting Spearman’s correlation analysis. Additionally, logistic regression models incorporating weight or BMI combined with age were performed to determine whether weight or BMI contributed to exposure differences after accounting for age effects. *p <* 0.05 was considered to show statistical significance.

## 3. Results and Discussion

### 3.1. Method Validation

The calibration equation for DA using the described method was y = 1.0542x + 0.1163, demonstrating excellent linearity (R^2^ = 0.9995). The LOQ and LOD for DNS-DA in urine were identified as 0.10 and 0.087 ng/mL, respectively. Recovery measured in artificial urine was 71 ± 6%, with all calibration standard solutions and blanks processed in the same batch as the real samples. No target compounds were detected in the blank solutions.

**Figure 2:**
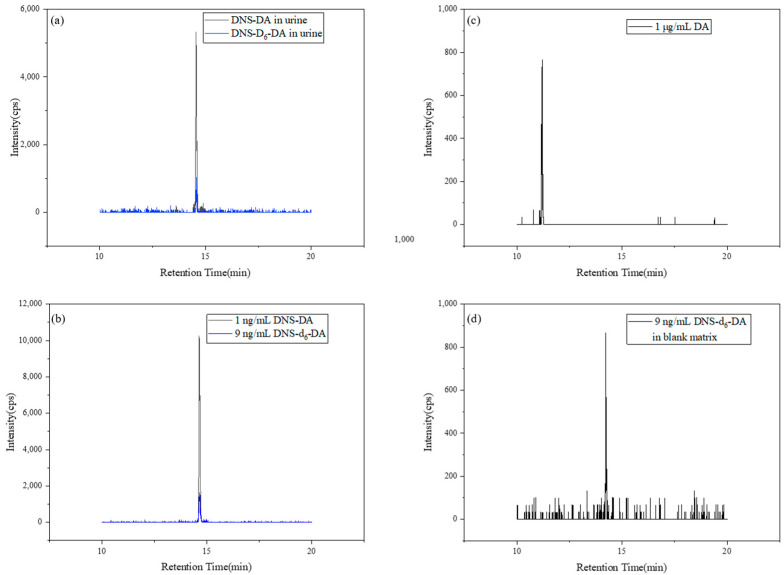
HPLC–MS/MS total-ion chromatograms for DNS-DA analysis. (**a**) Detection of DNS-DA and DNS-d_6_-DA in representative urine sample; (**b**) detection of 1 ng/mL standard DNS-DA and 9 ng/mL DNS-d_6_-DA; (**c**) detection of the 1 μg/mL DA; (**d**) blank matrix sample spiked with 9 ng/mL DNS-d_6_-DA.

The ME was calculated to be 90%, indicating slight suppression of HPLC–MS/MS signals for target compounds. To mitigate this effect, the isotopic tracer DNS-d_6_-DA was employed. As illustrated in Figure 2, endogenous chemicals and xenobiotics did not significantly influence test results, suggesting good analytical specificity for real urine samples. Data ranged from 85 to 115%, demonstrating that urine matrix effects were well-controlled [29].

Figure 2 illustrates that dansylation significantly enhanced sensitivity, with DNS-DA showing 1 × 10^4^-fold more sensitivity than unmodified DA in HPLC–MS/MS analysis. This improvement stems from the reduced polarity and enhanced ionization efficiency, overcoming the high LOQ of DA and enabling accurate quantification of endogenous DA at low concentrations. The strategy clearly outperformed direct DA analysis and demonstrated superior analytical performance over conventional direct DA detection approaches.

### 3.2. Levels of DA in Urine Samples

As shown in Table 1, the detect frequency (DF) of DA in total urine samples was 87.0%, indicating widespread low-dose exposure in children and adolescents in southern China. Quantifiable DA concentrations in the 216 pediatric participants ranged from non-detectable levels (<LOD) to 12.61 ng/mL, and the median concentration was 2.17 ng/mL.

These findings align with environmental monitoring data from Chinese coastal waters, where DA has been commonly detected at concentrations below regulatory thresholds [30,31]. In the Daya Bay region of the South China Sea, plankton samples exhibited an exceptionally high DA detection frequency of 98.3%, with concentrations reaching 5340 µg/kg (wet weight) [32]. Similarly, molluscan species from the Beibu Gulf showed a 17.7% detection frequency, with maximum concentrations of 401 µg/kg (wet weight) in contaminated specimens [33]. The human exposure profile observed in our study demonstrates geospatial consistency with environmental contamination patterns documented in adjacent coastal ecosystems. While this geospatial consistency is notable, methodological constraints affect urinary DA interpretation: Single urine collections capture momentary exposure snapshots that may miss intra-individual temporal dynamics. Hydration status and acute dietary shifts complicate spot biomarker reliability.

### 3.3. Effect of Gender

We analyzed samples by gender, including 113 males and 103 females. The detection frequency (DF) in males was 90.3%, slightly higher than that in females (83.5%). The median DA concentration in male samples was 2.44 ng/mL, also marginally higher than in females (2.00 ng/mL). However, the Mann–Whitney U test indicated no statistically significant difference in urinary DA concentrations between males and females (*p >* 0.05), suggesting comparable exposure levels regardless of gender. To date, no studies have examined the relationship between DA concentration and gender in mammals. Interestingly, one study in octopuses observed a decreasing trend in DA concentration with maturity in females, but not in males [34]. The absence of gender-related differences in our human cohort may reflect species-specific variations in DA metabolism between humans and cephalopods.

### 3.4. Effect of Age

**Figure 3:**
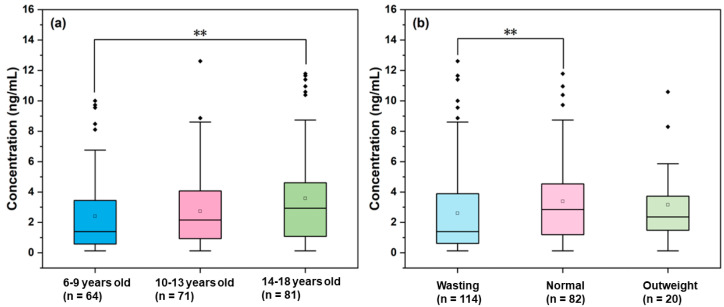
Urinary DA concentrations classified by (**a**) age groups and (**b**) BMI groups. In both panels, boxes represent the 25th and 75th percentiles, whiskers indicate the 10th and 90th percentiles, and the line within each box denotes the median. ** indicates *p* < 0.01.

**Figure 4:**
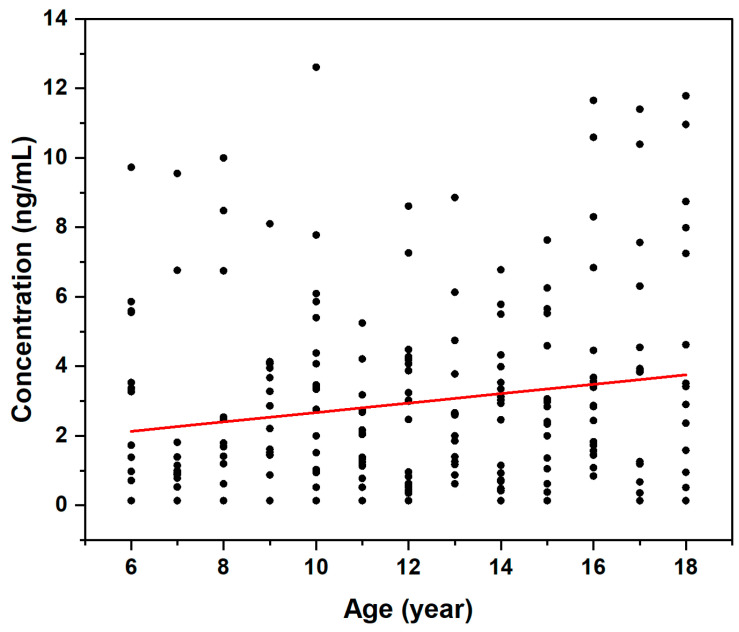
Linearity of urinary levels of DA with age.

DA concentrations were categorized into three groups according to age: 6–9 years (*n* = 64), 10–13 years (*n* = 71), and 14–18 years (*n* = 81). DF was lowest in the children aged 6 to 9 years (76.6%), while those aged 10 to 13 years and 14 to 18 years had higher DFs of 91.6% and 91.4%, respectively. Significant differences in urinary DA concentration were observed among the age groups (*p <* 0.05), with the median concentrations 1.40 ng/mL (6 to 9 years), 2.16 ng/mL (10 to 13 years), and 2.93 ng/mL (14–18 years) (Figure 3). As shown in Figure 4, Spearman’s correlation analysis demonstrated that age was positively linked to DA concentration (r = 0.183, β = 0.157, *p* = 0.07). The coefficient of determination (R^2^ = 0.37), indicated that age explained 37% of the variation in DA levels. These findings demonstrated an age-dependent trend in exposure to urinary DA in children and adolescents from southern China. This age-related pattern may be attributed to several factors. It has been shown that seafood consumption is positively correlated with age [35], which may lead to increased exposure to it. It is also known that DA is eliminated almost exclusively by renal mechanisms [36] and has a low absolute bioavailability (only 7 ± 4% in monkeys) [37]. Thus, repeated low-dose exposure to contaminated seafood may result in progressive bioaccumulation, explaining the elevated urinary DA concentrations observed in older adolescents. This study is the first to report the association between DA exposure levels and age across any biological taxa.

### 3.5. Effect of BMI

We categorized the DA concentration data in accordance with BMI status: normal (BMI between 18.5 and 25 kg/m^2^, *n* = 82), wasting (BMI less than 18.5 kg/m^2^, *n* = 114), and overweight (BMI greater than 25 kg/m^2^, *n* = 20). The median concentrations of the three data groups were 2.84 ng/mL (DF = 76.3%), 1.39 ng/mL (DF = 100%), and 2.36 ng/mL (DF = 95.0%), respectively. Significant differences in DA concentration between the three groups were observed (*p <* 0.05). We considered that the urine DA concentration followed the order normal > overweight > wasting (Figure 3). However, no linear correlation was found between the concentration of DA and BMI from 11.90 to 31.82 kg/m^2^. The lowest urinary DA concentrations in the wasting group may be due to malnutrition, which is associated with lower BMI [38], potentially resulting in consuming less seafood. Interestingly, we observed higher urinary DA concentrations in the normal than in the overweight group. Although this result may reflect variations in DA metabolism or excretion related to BMI, the underlying mechanisms remain to be considered. Potential associations of this finding with overweight (e.g., changes in metabolism or dietary habits that are dependent on body composition) should be explored in further studies.

To our knowledge, no previous studies have examined the relationship between the urine concentrations of DA and BMI in humans. Limited research in marine organisms has shown mixed results: DA in the *Emerita analoga* was found to be independent of weight [39], while the concentration of DA was found to be negatively correlated with the weight of the female octopus [34]. The differences among our findings and other studies in marine organisms may be due to a fundamental discrepancy in absorption and metabolism between mammals and non-mammalian species.

### 3.6. Estimated Daily Intake and Risk Assessment

Different exposures were identified for risk assessment based on age-specific average urine volumes and participants’ body weight, accounting for the lowest and highest levels of DA detected in urine. As shown in Table 1, the EDI of DA among children and adolescents ranged from 1.73 to 374 ng/kg/day. A significant difference was observed in the 10–13 years group (median: 36.7 ng/kg/day; range: 2.16–374 ng/kg/day) and 14–18 years group (median: 55.9 ng/kg/day; range: 2.21–244 ng/kg/day) (*p <* 0.05), while no difference was observed among other age groups, BMI groups and gender groups (*p >* 0.05). Through the logistic regression model analyses, we observed that adolescents aged 14–18 years had 2.25 times the risk of higher DA concentrations when compared with children aged 6–9 years. This finding suggests that older adolescents had a higher risk of DA exposure. Although no gender differences in urinary DA concentrations were found, males may face greater health risks. This is because males exposed to low levels of DA may show higher susceptibility to serious neurotoxicity than females [22].

DA has been commonly detected in marine algae and shellfish in southern China [40]. Because of regional dietary habits, seafood likely represents a major exogenous source of DA exposure for children and adolescents [41]. However, describing their seafood consumption accurately was challenging because they were unfamiliar with seafood and could not easily distinguish it from other aquatic products. The questionnaire used in this survey failed to find differences in participants’ diets. Nevertheless, the quantification of dietary intake faced substantial methodological hurdles. Key constraints in exposure assessment are worth considering. Reliance on parent/child hindsight recall tools introduces inherent uncertainty, especially given the fragmented nature of dietary sources—most students in China will consume lunch at school and breakfast and dinner at home, and some even consume both lunch and dinner at school—blurring precise attribution of intake. Inaccurate recall and inconsistent reporting between guardians and students are factors that may reduce the reliability of exposure indicators. As a result, subjects had difficulty distinguishing seafood subcategories, which, combined with these systematic assessment deficiencies, limited the ability of the questionnaire to discern dietary patterns.

Currently, no regulatory agency has established a tolerable daily intake of DA. Studies on the risk of low doses of exposure in rats have shown that administrating DA (2 mg/kg bw) intraperitoneally impaired their spatial memory [42]. Studies of long-term, low-dose exposure in rats demonstrated that single or repeated doses of DA (0, 1 or 2 mg/kg bw) administration did not produce adverse effects [43]. The EDI estimated in our study were all below the DA dose that may cause neurotoxicity in rats. As previously described in Section 3.2, the DA concentrations detected in shellfish in Southern China [32,33] were 1–2 orders of magnitude below current regulatory limits (20 mg/kg) [18]. These results suggest that the risk of DA exposure among children and adolescents in Southern China was within a manageable safety range with no risk of acute exposure. Nevertheless, numerous laboratory studies have shown that even at low doses, DA can produce adverse effects such as neurotoxicity and the induction of seizures [44]. Therefore, further investigation of DA exposure is warranted in future studies.

## 4. Conclusions

This study employed the DNS-Cl derivatization method to quantify urinary DA concentrations in 216 children and adolescents from southern China, establishing critical baseline data and analyzing factors that may influence exposure levels. The high detection rate (87%) indicates that DA exposure is widespread among children and adolescents in this region. Significant differences in urinary DA concentrations were observed across age and BMI groups, while no gender-related differences were detected. Notably, a positive correlation between urinary DA concentration and age suggests that DA exposure increases with age. All EDIs in this study were below the threshold for neurotoxicity, though older adolescents and males may face relatively higher risk profiles. These findings underscore the importance of comprehensive studies to investigate the health effects of long-term, low-dose DA exposure in different populations and to develop more robust risk assessment frameworks and management strategies.

Several limitations of this study should be acknowledged. First, dietary exposure assessment relied on retrospective questionnaires completed jointly by parents or guardians and students. The complexity of meal sources—where lunch is typically provided by schools and breakfast and dinner are prepared at home—introduces uncertainty in accurately attributing dietary intake. This, combined with potential recall bias and inconsistencies between parent and student reporting, may affect the precision of exposure estimation.

Second, the cross-sectional design and reliance on single spot urine samples may not fully capture temporal variability in DA exposure. Urinary biomonitoring, although non-invasive and practical, is subject to fluctuations due to hydration status, diurnal variation, and short-term dietary changes, which can impact the reliability of exposure assessment. Additionally, the study focused solely on quantifying DA itself, without measuring potential urinary metabolites or additional biomarkers related to neuroinflammation or oxidative stress, which could provide deeper mechanistic insights.

Finally, the absence of concurrent clinical assessments—such as neurocognitive testing or neuroimaging—limits the ability to directly correlate exposure levels with health outcomes. The relatively modest sample size and regional focus may also limit the generalizability of the findings to broader populations.

Despite these limitations, this study provides essential baseline data for DA exposure in children and adolescents in southern China and highlights the need for future longitudinal and multi-center studies. Future research should incorporate more comprehensive dietary assessments, repeated biomonitoring, and clinical evaluations to better elucidate the health impacts of chronic, low-dose DA exposure and to inform targeted risk management strategies.

## Figures and Tables

**Table 1:** Urinary concentration and daily intake of DA of children and adolescents in southern China.

Groups		*n*	*n* (<LOD)	Concentration (ng/mL)		DF	EDI (ng/kg/day)
				Median	25th–75th Percentiles		
All		216	28	2.17	0.87–4.08	87.0%	1.73–374
Gender	Male	113	11	2.44	1.03–4.07	90.3%	1.73–374
	Female	103	17	2.0	0.6–4.19	83.5%	2.16–321
Age (years)	6–9 years	64	15	1.40	0.55–3.49	76.6%	1.73–321
	10–13 years	71	6	2.16	0.94–4.07	91.6%	2.16–374
	14–18 years	81	7	2.93	1.06–5.06	91.4%	2.21–244
BMI (kg/m^2^)	≤18.5	114	27	1.39	0.61–3.90	76.3%	2.65–374
	18.5–25	82	0	2.85	1.18–4.56	100%	2.16–307
	≥25	20	1	2.36	1.44–3.75	95.0%	1.73–141

LOD = limit of detection; DF = detect frequency; BMI = body mass index; EDI = cumulative daily intake.

## Data Availability

The raw data supporting the conclusions of this article will be made available by the authors on request.

## Abbreviations

The following abbreviations are used in this manuscript:

DA	Domoic acid
DNS-Cl	Dansyl-chloride
HPLC–MS/MS	High-performance liquid chromatography–tandem mass spectrometry
EDI	Estimated daily intake
DF	Detect frequency
BMI	Body mass index
LOD	Limit of detection
LOQ	Limit of quantification
IQR	Interquartile range
